# Synthesis of Hantzsch Adducts as Cholinesterases and Calcium Flux inhibitors, Antioxidants and Neuroprotectives

**DOI:** 10.3390/ijms21207652

**Published:** 2020-10-16

**Authors:** Irene Pachón Angona, Helene Martin, Solene Daniel, Ignacio Moraleda, Alexandre Bonet, Artur Wnorowski, Maciej Maj, Krzysztof Jozwiak, Isabel Iriepa, Bernard Refouvelet, José Marco-Contelles, Lhassane Ismaili

**Affiliations:** 1 Neurosciences Intégratives et Cliniques EA 481, Pôle de Chimie Organique et Thérapeutique, Univ. Bourgogne Franche-Comté, UFR Santé, 19, rue Ambroise Paré, F-25000 Besançon, France; pachon.angona.irene@gmail.com (I.P.A.); solene.du70@hotmail.fr (S.D.); 2 PEPITE EA4267, Laboratoire de Toxicologie Cellulaire, Univ. Bourgogne Franche-Comté, F-25000 Besançon, France; helene.martin@univ-fcomte.fr (H.M.); alexandre.bonet@univ-fcomte.fr (A.B.); 3 Department of Organic Chemistry and Inorganic Chemistry, School Sciences, University of Alcalá, Ctra. Barcelona, Km. 33.6, 28871 Alcalá de Henares, Spain; ignacio.moraleda@uah.es; 4 Department of Biopharmacy, Medical University of Lublin, ul. W. Chodzki 4a, 20-093 Lublin, Poland; artur.wnorowski@gmail.com (A.W.); maciejmaj@umlub.pl (M.M.); krzysztof.jozwiak@umlub.pl (K.J.); 5 Laboratory of Medicinal Chemistry (IQOG, CSIC), Juan de la Cierva, 3, 28006-Madrid, Spain; iqoc21@iqog.csic.es

**Keywords:** Alzheimer’s disease, Ca^+2^ channel antagonists, Hantzsch reaction, multitarget directed ligands, neuroprotection, oxidative stress

## Abstract

We report herein the design, synthesis, biological evaluation, and molecular modelling of new inhibitors of acetylcholinesterase (AChE) and butyrylcholinesterase (BuChE), able to block Ca^+2^ channels also showing antioxidant and neuroprotective activities. The new MTDL, dialkyl 2,6-dimethyl-4-(4-((5-aminoalkyl)oxy)phenyl)-1,4-dihydropyridine-3,5-dicarboxylate **3a-p**, have been obtained via Hantzsch reaction from appropriate and commercially available precursors. Pertinent biological analysis has prompted us to identify MTDL **3h** [dimethyl-4-(4-((5-(4-benzylpiperidin-1-yl)pentyl)oxy)phenyl)-2,6-dimethyl-1,4-dihydropyridine-3,5-dicarboxylate] as an attractive inhibitor of AChE (1.8 μM) and BuChE (2 μM), Ca^+2^ channel antagonist (47.72% at 10 μM), and antioxidant (2.54 TE) agent, showing significant neuroprotection 28.68% and 38.29% against H_2_O_2,_ and O/R, respectively, at 0.3 μM, thus being considered a hit-compound for further investigation in our search for anti-Alzheimer’s disease agents.

## 1. Introduction

Alzheimer’s disease (AD) is the most common dementia, mainly affecting the elderly population. It causes the progressive loss of neurons, leading to an impairment of the cognitive function and a loss behavioral abilities [1]. Although its etiopathology remains unclear, many risk factors appear to increase the susceptibility and progression of this disorder, such as diet, environment and or genetics.

AD is histologically characterized by the presence of amyloid plaques and neurofibrillary tangles (NFTs), mainly consisting of accumulations and depositions of *β*-amyloid (A*β*) peptide and hyperphosphorylated tau protein (pTau) [2]. Other physiopathological features include synaptic deterioration and disbalances of Ca^+2^ ions and biometals, which in turn cause oxidative stress. They are all interconnected, resulting in a progressive and irreversible neurodegeneration leading to cell damage and neuronal death [2].

To date, the only available therapy for this disorder is symptomatic and is far from stopping, slowing or reversing the progression of AD. Hence, there is a need for developing new and more efficient therapies [3,4]. The traditional mono-target approach is not adapted to such a multifactorial disease. In response to this, a more recent method called multi-targeted directed ligands (MTDLs) has emerged. These ligands are molecules bearing different pharmacophores in just one molecule, that are capable to act simultaneously on several etiopathological AD features. Accordingly, several MTDLs have been developed by many research groups [5,6,7,8]. In the last decade, we have contributed to this area by using multicomponent reactions (MCR) [9,10,11,12,13] as a very suitable and efficient synthetic tool, with its easy performance and its time-saving procedure leading a higher structural diversity.

In this context, we report herein the design, synthesis and biological evaluation of new MTDL as inhibitors of acetylcholinesterase (AChE) and butyrylcholinesterase (BuChE), able to lower the oxidative stress (OS), and block Ca^+2^ channels. In fact, it is well known that AChE inhibitors (AChEIs) not only facilitate cholinergic transmission, but also interfere with the synthesis, deposition and aggregation of toxic A*β*. [14,15] In addition, other studies suggested that BuChE may also influence the aggregation of A*β* into neuritic plaques and formation of the NFT deposit. [16,17]. Otherwise, oxidative stress (OS) caused by various underlying factors, such as mitochondrial dysfunction, and disruption of metal homeostasis (Cu, Fe, Zn), plays a crucial role in the pathogeny of AD. Therefore, it is evident that the antioxidant strategy constitutes one of the most promising pathways for the development of new drugs for aging-related diseases [18]. Ca^+2^ channel blockade is also one of the promising pathways in the development of new drugs for AD, as evidenced by the example of nivaldipine, currently under clinical development [19]. The increase in cytosolic calcium levels causes mitochondrial disruption, which leads to the activation of the apoptotic cascade and cell death [20], and potentiates NFT formation [21].

## 2. Results

### 2.1. Synthesis

**Scheme 1:**
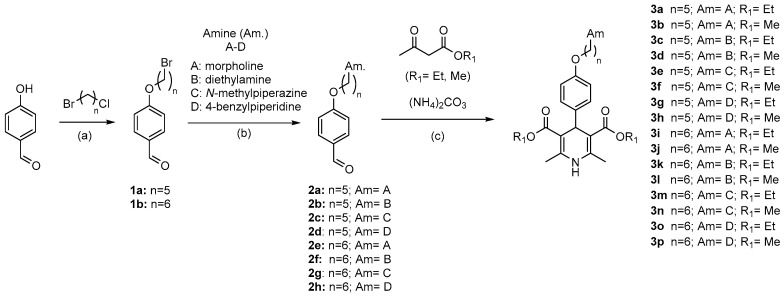
Synthesis of dialkyl 2,6-dimethyl-4-(4-((5-aminoalkyl)oxy)phenyl)-1,4-dihydropyridine- 3,5-dicarboxylate **3a-p**. Reagents and conditions: (**a**) K_2_CO_3_, acetonitrile, 18 h, reflux; (**b**) K_2_CO_3_, acetonitrile, 24 h, reflux; (**c**) EtOH/H_2_O, O.N., 75 °C.

The synthesis of the new MTDL 3a-p has been carried out in one-pot Hantzsch reaction of aldehydes **2-h** with ethyl or methyl acetoacetate and ammonium carbonate, in EtOH/water (Scheme 1). Aldehydes **2-h** were synthesized in two steps. In the first step, the alkyl chain (*n*= 5 and *n*= 6) was incorporated by nucleophilic substitution on the hydroxyl group at the C4 of the 4-hydroxybenzaldehyde in the presence of K_2_CO_3_ in acetone to afford intermediates **1a-b**. Then, in the second step, compounds 1a-b were reacted with different amines, such as morpholine, diethylamine N-methylpiperazine and 4-benzylpiperidine. All new compounds showed excellent analytical and spectroscopic data, in good agreement with the expected values (see Material and Methods).

To verify the effectiveness of our design, compounds 3a-p were submitted to AChE, BuChE inhibition assay, Ca^+2^ channel blockade, antioxidant evaluation, followed by the neuroprotection analysis of selected compounds.

### 2.2. Biological Evaluation

#### 2.2.1. Inhibition of EeAChE/eqBuChE

For the ChE inhibition experiments, we used the Ellman protocol [22], the cheap and easily available EeAChE and eqBuChE and tacrine as reference. As shown in Table 1, only compound 3h had a percentage of inhibition against AChE at 10 mM higher than 50% and showed an IC_50_ equal to 1.8 μM, only 60-fold less active than tacrine (IC_50_ = 0.03 μM) [23,24]. However, five compounds 3c, 3h and 3k-l were active against BuChE with IC_50_ ranging from 2.0 μM for 3h to 9.6 μM for 3k. Concerning the structure activity relationship (SAR), the diethylamine moeity seems to be favorable, regardless of the length of the linker, but no additional clear SAR could be established.

#### 2.2.2. Ca^+2^ Channel Blockade

Ca^+2^ channel blockade capacity of compounds 3a-p, compared to nimodipine as standard, has been carried out following the usual methodology [12,13,25]. The new compounds showed a good inhibition percentage of calcium flux ranging from 18.58 (3a) to 80.19 (3o). The most potent compounds corresponded, in increasing order, to 3i (47.55), 3h (47.72), 3m (50.6), 3j (52.57), 3c (58.21), 3g (53.6), 3p (78.13) and 3o (80.19) comparing thus very favorably with nimodipine (47.91). It is worth mentioning that compounds 3c, 3h and 3m are also the potent ChEI and are therefore the most balanced compounds in the two assays. From the point of view of SAR, compounds with N-methylpiperazine and N-benzylpiperidine moieties showed better results with compounds bearing ethylester than methylester groups, whatever the length of the linker. Except for the compounds bearing the methylamine group, all compounds with the ethyester group showed better inhibition when the length of the linker is equal to six than those with a linker with five carbons.

#### 2.2.3. Antioxidant Analysis

The antioxidant activity expressed as Trolox equivalents (TE) units of compounds 3a-g, compared to melatonin (MEL), used as positive control, with ORAC value 2.45 [9], was determined by the ORAC-FL method [26]. As shown in Table 1, the values for the antioxidant capacity range from 0.91 (3l) to 2.64 (3c). Three compounds, 3b (2.59) and two of the most balanced compounds, 3c (2.64 TE) and 3h (2.54 TE), showed antioxidant activities higher than melatonin (2.45 TE). The antioxidant power of the third balanced compound 2m displayed only 1.10 TE, thus representing half of the activity of melatonin. No evident SAR could be drawn for these results

#### 2.2.4. Neuroprotective Activity

The most balanced compounds were then submitted to evaluation of their neuroprotection activity. For this purpose, cytotoxicity was induced by H_2_O_2_, a well-known toxic responsible for the generation of ROS and by mitochondrial respiratory chain blockers oligomycin rotenone (O/R). Prior to the neuroprotective assay, the effect of the compounds on cell viability was evaluated at 1 and 10 µM, showing no cytotoxicity against SH-SY5Y cells. As shown in Table 2, compounds 3c and 3m displayed a modest neuroprotective effect against H_2_O_2_ and O/R. Nevertheless, and very interestingly, compound 3h showed an interesting effect against H_2_O_2_, particularly at 0.3 µM, where it showed a percentage of neuroprotection equal to 28%. This neuroprotective positive effect is more pronounced against O/R with a percentage equal to 38.29 and 33.90 at 0.3 and 1 µM, respectively, compared to melatonin with a percentage equal to 66.6 at 0.1 µM.

### 2.3. Molecular Docking Studies

The potential binding mode of the most active compound, **3h**, with hAChE (PDB: 1B41) and hBChE (PDB: 4BDS) was investigated by molecular docking, which was performed by using Autodock Vina [27] with structure images created by Discovery Studio. As we previously described [25], conformational flexibility of AChE was considered by allowing eight side chains to be flexible during the docking process.

**Figure 1:**
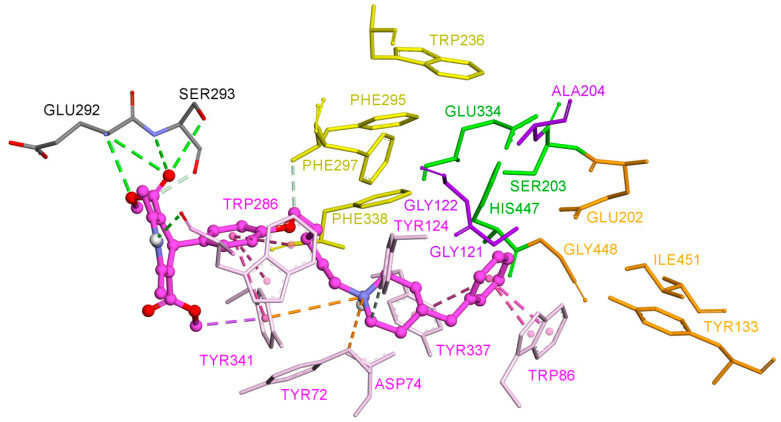
**Binding mode of 3h in the active site of hAChE.** Compound **3h** is rendered as ball and stick (carbon atoms in pink) and the side chain conformations of the mobile residues are illustrated in light pink. The catalytic triad (CT) is colored in green, oxyanion hole (OH) in magenta, anionic subsite (AS) in orange, except Trp86, acyl binding pocket (ABP) in yellow and peripheral anionic site (PAS) in light pink. Hydrophobic contact, π–π stacking and π–π T-shaped were depicted in purple dot lines, H-bonds were in dark green dot lines, attractive charge and π–cation in orange and carbon hydrogen bond in light green dot lines.

**Figure 2:**
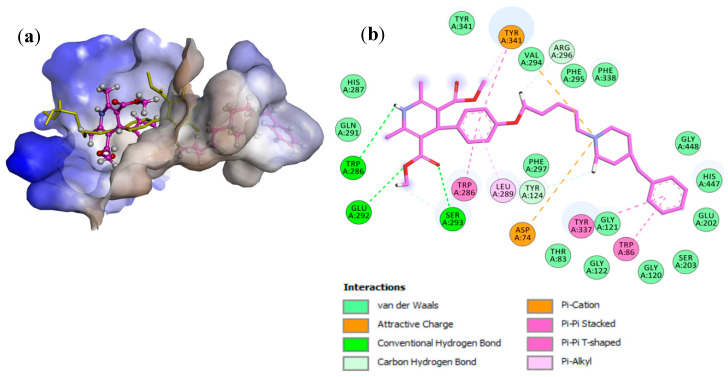
(**a**) Three-dimensional surface representation of hAChE with compounds **3h** (pink) and **3d** (yellow). (**b**) Two-dimensional schematic view of the interactions between **3h** and hAChE.

The results show that **3h** was quite strongly bound with the optimal conformation of AChE, and its binding energy reached −10.3 kcal/mol. The whole molecule of **3h** entered the binding pocket of AChE in a linear mode, and the benzyl piperidinium moiety was inserted into the interior of the pocket (Figure 1 and Figure 2a). Due to the large molecular size, it could completely occupy the entire cavity (Figure 2a) and the structure of one of the methoxycarbonyl groups on the dihydropyridine ring extends more outwardly, forming four hydrogen bonds with Ser293 and Glu292 (Figure 1). At the bottom of the gorge, the phenyl ring interacted with Trp86 (anionic subsite, AS) and Tyr337 via π–π stacking interactions, and this ring was positioned to favor van der Waals interactions with the catalytic triad (CT) residues His447 and Ser203. The protonated piperidine moiety interacted with Asp74 and Tyr341, in the peripheral anionic site (PAS), via attractive charge and π–cation interactions, respectively. The pentamethylene chain interacted halfway down the gorge, with the acyl binding pocket (ABP, Phe295, Phe297 and Phe338). The phenoxy ring interacted with Trp286 and Tyr341, in the PAS, via π–π T-shaped interactions. At the mouth of the gorge, the NH dihydropyridine group also formed a hydrogen bond with Trp286 (Figure 2b).

Docking experiments of a non-active compound, **3d**, was then performed to predict why the other dihydropiridines do not bind well to AChE. It showed that compound **3d** has much lower affinity for hAChE (binding energy, −6.8 kcal/mol) than compound **3h**. This compound is accommodated in PAS and no interaction with the catalytic triad aminoacids (His447, Ser203 and Glu334) has been established. Contrary to compound **3h**, the dihydropyridine moiety is oriented to the interior of the pocket and it forms two hydrogen bonds between the NH group and two aminoacids of the PAS (Tyr124 and Asp74). Additionally, the phenyl ring forms π–π T-shaped interaction with Trp286 (PAS). The diethylamonium group extends outside the gorge interacting with His287 and Gln291.

**Figure 3:**
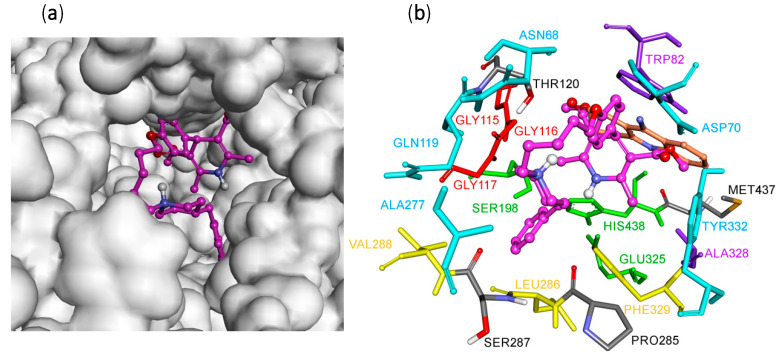
Docking pose of compound **3h** and tacrine inside gorge cavity of hBuChE. (**a**) Top view of the accessible surface of the active site gorge (white), compound **3h** is rendered in pink ball and stick model. (**b**) Compound **3h** was shown in pink, tacrine in brown, catalytic anionic site (CAS) in green, OH in red, choline binding site (CBS) in violet, ABP in yellow, and PAS in blue.

Analysis of the binding mode of the compound **3h** inside the active site of BChE revealed that the compound stabilized itself inside the pocket through entangling several interactions (docking score = −9.7 kcal/mol), adopting an U-shaped conformation (Figure 3a).

**Figure 4:**
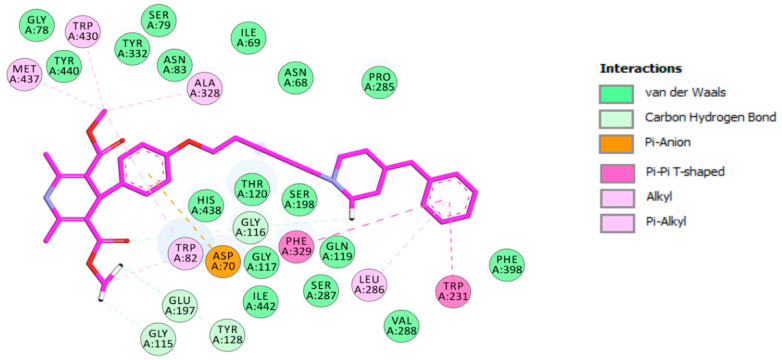
Two-dimensional schematic view of the interactions between **3h** and hBChE.

The protein complex with the best-docked pose of compound **3h** showed that both carboxylate groups of the docked ligand were superimposed on the native co-crystallized tacrine (Figure 3b). In this position, the dihydropyridine moiety is oriented toward the bottom of the active site, as with tacrine, and it binds in the catalytic anionic site (CAS) region of the enzyme, establishing π–alkyl, alkyl and carbon hydrogen interactions with many amino acids, including two key amino acids of the choline binding site (CBS) pocket (Trp82 and Ala328). The phenoxy–alkyl linker chain went from the bottom to the mouth of the gorge, allowing the benzyl–piperidinium moiety to enter the cavity again. In this situation, π–anion interaction is established between Asp70 (peripheral anionic site, PAS) and the phenoxy ring. In addition, the alkyl chain also interacts with Asn68 (PAS) via van der Waals contacts (Figure 4).

The piperidinium ring, inside the cavity, pointed toward the catalytic triad (CT) amino acid residues (Glu325, His438, and Ser198), causing several methylene groups to establish van der Waals interactions with the last two amino acids. On the other hand, methylene groups can also interact with Gly116 and Gly116 (oxyanion hole, OH) and Pro285 (located near the acyl pocket, ABP) (Figure 4).

Finally, the terminal phenyl group of the compound, going down, anchored inside the active site gorge by establishing two π–π interactions with Trp231 (located near the ABP) and Phe329 (ABP) (Figure 4).

**Figure 5:**
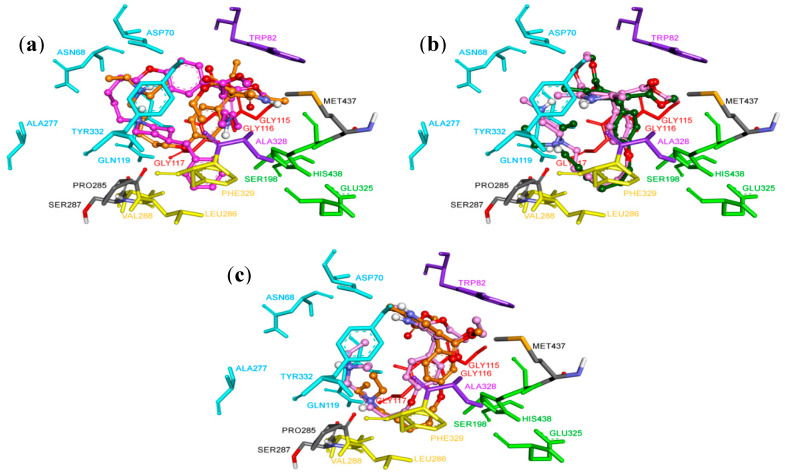
Docking pose of compounds **3c**, **3d**, **3h** and **3k-3m** inside gorge cavity of hBuChE. (**a**) Compound **3h** is shown in pink, compound **3c** in orange. (**b**) Compound **3l** is shown in green, compound **3m** in light violet. (**c**) Compound **3d** is shown in orange, compound **4k** in light pink. All compounds are represented in ball and stick models, CAS in green, OH in red, CBS in violet, ABP in yellow, and PAS in blue.

**Figure 6:**
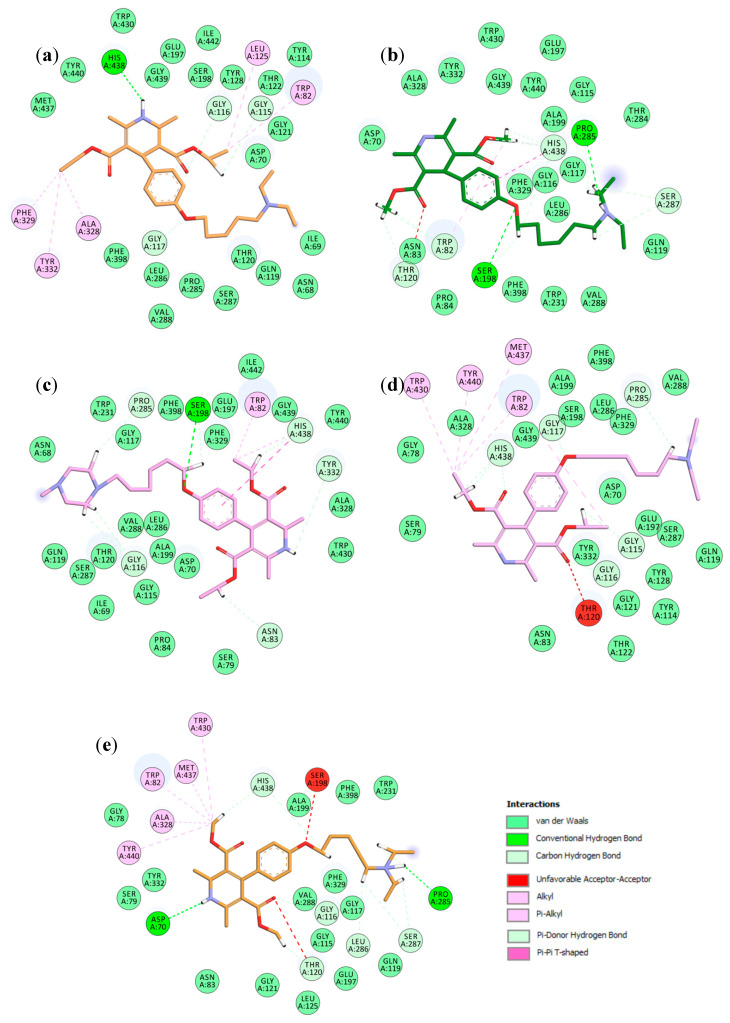
Two-dimensional schematic view of the interactions between **3c** (**a**), **3l** (**b**), **3m** (**c**), **3k** (**d**), **3d** (**e**) and hBChE.

The docking results also reveal that BuChE could effectively accommodate compound **3c** inside the active site gorge. The ligand is placed into the binding pocket in a similar way to compound **3h** (docking score = −8.7 kcal/mol) (Figure 5a). In this situation, one carboxylate group and the dihydropyridine moiety of the docked ligand were superimposed onto the native co-crystallized tacrine. The NH group, oriented toward the bottom of the active site, forms a hydrogen bond and van der Waals interaction with the catalytic triad residues His438 and Ser198, respectively. Moreover, methyl of the carboxylate groups interacts with Trp82 and Ala328 (CBS pocket), with Phe329 (ABP) and with Tyr332 (PAS). The carboxylate group also interacts with Gly115 and Gly116 (OH) (Figure 6a).

The estimated binding energies for BuChE-**3l** and BuChE–**3m** complexes are −7.8 and −7.9 kcal/mol. Both compounds showed a similar disposition inside the cavity, especially with regard to the dihydropyridine moiety (Figure 5b). Contrary to compounds **3h** and **3c**, the NH group points to the mouth of the gorge, placing the phenoxy–alkyl linker deep inside the cavity. Hydrogen bond was observed between the oxygen of phenoxy group of **3l** and **3m** with Ser198 (catalytic triad). Apart from this, His438 established π–π T-shaped interaction with the phenyl ring, π–alkyl and carbon hydrogen interactions with the alkyl group of one carboxylate moiety. Residues such as Trp82 (CBS pocket), Tyr332 and Thr120 (PAS) were also involved in other non-polar interactions (Figure 6b,c).

The less active compound againts BuChE, **3k**, was fitted on the enzyme in a mode similar to that of the non-active compound (**3d**) (Figure 5c**)** but there was difference between the energy of interaction of the selected poses for both compounds (**3k**: −7.5 kcal/mol, **3d**: −6,8 kcal/mol). The NH group is pointed toward the entrance of the cavity, which placed the phenoxy–alkyl linker and one carboxylate group in the bottom of the gorge. The alkyl group of the carboxylate moiety formed carbon hydrogen bonds with His438 and a network of alkyl and π–alkyl interactions with Trp82, Met437 and Tyr440 (Figure 6d,e). Both compounds showed an unfavorable acceptor-acceptor interaction with Thr120 but the non-active compound (**3d**) exhibited another such interaction with one amino acid of the catalytic triad (Ser198) (Figure 6c).

## 3. Materials and Methods

### 3.1. Chemistry

#### 3.1.1. General Methods

All reagents were purchased from Sigma Aldrich or TCI. Progress of the reactions was monitored with TLC using aluminum sheets with silica gel 60 F254 from Merck (Kenilworth, NJ, USA). ^1^H and ^13^C NMR spectra were recorded on a Bruker spectrometer, operating at 400 and 100 MHz, respectively, using CDCl_3_ as a solvent. The chemical shifts are reported in parts per million (ppm), using tetramethylsilane (TMS) as internal reference. The multiplicities of the signals are indicated by the following abbreviations: s, singlet; d, doublet; t, triplet; q, quadruplet; and m, multiplet coupling constants, and they are expressed in Hz. High resolution mass spectra were obtained at Centre Commun de Spectrométrie de Masse, Lyon, France on a Bruker micrOTOF-Q II spectrometer (Bruker Daltonics) in positive ESI-TOF (electrospray ionization-time of flight). Elemental analyses were obtained by a Carlo Erba EA 1108 analyzer, and the analytical results are within ±0.2% of the theoretical values for all compounds.

#### 3.1.2. General Procedures for the Synthesis

**General procedure A**. 4-Hydroxybenzaldehyde (1 equiv) and the corresponding bromo-chloroalkane (1.5 equiv) were introduced with K_2_CO_3_ (3 equiv) in acetonitrile (1.7 mmol/mL). The reaction was stirred for 18 h at reflux. The crude oil was filtered over Celite^®^ and the filtrate was evaporated. The residue was filtered over silica with pentane to remove the excess of bromo-chloroalkane and finally with DCM to get the desired compound as an oil. The crude was then used without further purifications.

**General procedure B**. The corresponding halogenoalkoxy-benzaldehyde (**1a**-**b**) was introduced with the corresponding amine (**A**-**D**) (1.5 or 2 equiv.) and K_2_CO_3_ (3e quiv.) in acetonitrile (0.6 mmol/mL). The reaction was stirred for 24 h at reflux. The crude was filtered over Celite^®^ and the filtrate was evaporated. DCM was added to the filtrate and washed twice with water. The organic layers were dried with brine and over Na_2_SO_4_ to finally be purified by flash column chromatography.

**General procedure C.** 4-alkyloxy-benzaldehyde (**2a-h**) (1 equiv.) and the corresponding acetoacetate (3.5 equiv.) were dissolved in EtOH (4 mmol/mL). Successively, the same volume of H_2_O was added dropwise while sonicating the crude. The resulting mixture was stirred and heated at 75 °C for one hour. Finally, 2.5 equivalents of ammonium carbonate were added to the crude. The reaction was then stirred and heated at 75 °C overnight. The desired product precipitated once the crude reaction reached RT, or triturated in diethyl ether. The solid was then filtered and washed with diethyl ether again to finally afford the desired final adducts from 12 to 76% yields.

##### Synthesis of Compounds 1a-b

**4-((5-Bromopentyl)oxy)benzaldehyde (1a).** The crude was prepared according to **general procedure A** starting from commercially available 4-hydroxybenzaldehyde (1 equiv, 8.19 mmol, 1.00 g), 1-bromo-4-chloropentane (1.5 equiv, 12.28 mmol, 1.62 mL) and K_2_CO_3_ (3 equiv, 24.57 mmol, 3.40 g) in acetonitrile (25 mL) to afford 1.84 g of the desired compound as a transparent oil. The crude was used without further purification.

**4-((6-Bromohexyl)oxy)benzaldehyde (1b).** The crude was prepared according to **general procedure A** starting from commercially available 4-hydroxybenzaldehyde (1 equiv., 8.19 mmol, 1.00 g), 1-bromo-6-chlorohexane (1.5 equiv., 12.28 mmol, 1.83 mL) and K_2_CO_3_ (3 equiv., 24.57 mmol, 3.40 g) in acetonitrile (25 mL) to afford 1.57 g of the desired compound as a transparent oil. The crude was used without further purification.

##### Synthesis of Compounds 2a-h

**4-((5-Morpholinopentyl)oxy)benzaldehyde (2a).** The crude was prepared according to **general procedure B** starting from 4-((5-bromopentyl)oxy)benzaldehyde **1a** (1 equiv., 6.78 mmol, 1.84 g), commercially available morpholine (2 equiv., 13.56 mmol, 1.19 mL) and K_2_CO_3_ (3 equiv., 20.34 mmol, 2.81 g) in acetonitrile (25 mL) to afford 599.6 mg of the desired compound (32%) as a transparent oil. The crude was used without further purifications. ^1^H NMR (400 MHz, CDCl_3_) δ 9.88 (s, 1H), 7.89–7.75 (m, 2H), 7.05–6.94 (m, 2H), 4.05 (t, J = 6.4 Hz, 2H), 3.76 (s, 4H), *2.47 (d, J = 33.1 Hz, 6H), 1.94–1.77 (m, 2H), 1.62 (bs, 2H), 1.56*–1.45 (m, 2H). ^13^C NMR (101 MHz, CDCl_3_) δ 190.77, 164.15, 131.19, 129.82, 114.74, 68.97, 44.97, 32.48, 28.91, 26.61, 26.59, 25.34

**4-((5-(Diethylamino)pentyl)oxy)benzaldehyde (2b).** The crude was prepared according to **general procedure B** starting from 4-((5-bromopentyl)oxy)benzaldehyde **1a** (1 equiv., 6.78 mmol, 1.84 g), commercially available diethylamine (2 equiv., 13.56 mmol, 1.404 mL) and K_2_CO_3_ (3 equiv., 20.34 mmol, 2.81 g) in acetonitrile (25 mL) to afford 510.50 mg of the desired compound (29%) as a transparent oil. The crude was used without further purifications. ^1^H NMR (400 MHz, CDCl_3_) δ 9.87 (s, 1H), 7.85–7.78 (m, 2H), 7.03–6.92 (m, 2H), 4.03 (t, J = 6.5 Hz, 2H), 2.54 (q, J = 7.2 Hz, 5H), 2.45–2.36 (m, 2H), 1.82 (td, J = 13.3, 6.6 Hz, 3H), 1.56–1.43 (m, 4H), 1.39–1.31 (m, 2H), 1.03 (t, J = 7.2 Hz, 7H). ^13^C NMR (101 MHz, CDCl_3_) δ 190.91, 164.21, 131.99, 129.78, 114.75, 68.30, 52.80, 46.86, 29.09, 26.83, 24.10, 11.61.

**4-((5-(4-Methylpiperazin-1-yl)pentyl)oxy)benzaldehyde (2c).** The crude was prepared according to **general procedure B** starting from 4-((5-bromopentyl)oxy)benzaldehyde **1a** (1 equiv., 5.08 mmol, 1.38 g), commercially available N-methylpiperazine (2 equiv., 10.16 mmol, 1.13 mL) and K_2_CO_3_ (3 equiv., 15.23 mmol, 2.11 g) in acetonitrile (25 mL) to afford 415.6 mg of the desired compound (28%) as a transparent oil. The crude was used without further purifications. ^1^H NMR (400 MHz, CDCl_3_) δ 9.88 (s, 1H), 7.87–7.75 (m, 2H), 7.01–6.90 (m, 2H), 4.04 (t, J = 6.4 Hz, 2H), 2.52 (bs, 8H), 2.39 (dd, J = 17.5, 9.7 Hz, 2H), 2.31 (d, J = 9.9 Hz, 3H), 1.92–1.76 (m, 2H), 1.62–1.54 (m, 2H), 1.53–1.43 (m, 2H). ^13^C NMR (101 MHz, CDCl_3_) δ 190.81, 164.17, 132.00, 129.80, 114.97, 68.19, 58.49, 55.07, 53.18, 46.01, 28.97, 26.58, 24.00.

**4-((5-(4-Benzylpiperidin-1-yl)pentyl)oxy)benzaldehyde (2d).** The crude was prepared according to **general procedure B** starting from 4-((5-bromopentyl)oxy)benzaldehyde **1a** (1 equiv., 1.48 mmol, 403.00 mg), commercially available 4-benzylpiperidine (2 equiv., 2.97 mmol, 0.521 mL) and K_2_CO_3_ (3 equiv., 4.45 mmol, 651.00 mg) in acetonitrile (8 mL) to afford 257.00 mg of the desired compound (43%) as a transparent oil. The crude was used without further purifications. ^1^H NMR (400 MHz, CDCl_3_) δ 9.88 (s, 1H), 7.85–7.73 (m, 2H), 7.28 (d, J = 7.1 Hz, 2H), 7.19 (dd, J = 8.4, 6.3 Hz, 1H), 7.15–7.10 (m, 2H), 7.02–6.92 (m, 2H), 4.04 (t, J = 6.4 Hz, 2H), 3.04 (bs, 2H), 2.55 (d, J = 6.6 Hz, 2H), 2.45 (s, 2H), 1.99 (s, 2H), 1.88–1.79 (m, 3H), 1.68 (d, J = 11.4 Hz, 4H), 1.55–1.42 (m, 4H). ^13^C NMR (101 MHz, CDCl_3_) δ 190.82, 164.02, 140.70, 132.00, 129.79, 129.13, 128.16, 125.78,114.75, 68.22, 58.92, 54.02,, 43.22, 37.97, 43.22, 37.97, 32.13, 28.98, 26.75.

**4-((6-Morpholinohexyl)oxy)benzaldehyde (2e).** The crude was prepared according to **general procedure B** starting from 4-((6-bromohexyl)oxy)benzaldehyde **1b** (1 equiv., 2.02 mmol, 500 mg), commercially available morpholine (2 equiv., 4.04 mmol, 0.352 mL) and K_2_CO_3_ (3 Equiv., 6.06 mmol, 837.55 mg) in acetonitrile (20 mL) to afford 600 mg of the desired compound (48%) as a transparent oil. The crude was used without further purifications. ^1^H NMR (400 MHz, CDCl_3_) δ 9.88 (s, 1H), 7.86–7.80 (m, 2H), 7.06–6.95 (m, 2H), 4.04 (t, J = 6.4 Hz, 2H), 3.77 (s, 3H), 2.46 (d, J = 37.6 Hz, 6H), 1.82 (dd, J = 14.7, 6.7 Hz, 2H), 1.55–1.45 (m, 3H), 1.41 (dt, J = 8.7, 2.7 Hz, 2H), 1.25 (d, J = 4.9 Hz, 2H). ^13^C NMR (101 MHz, CDCl_3_) δ 190.80, 164.20, 131.99, 129.79, 114.74, 66.94, 59.03, 44.97, 32.48, 29.01, 26.78, 26.43, 25.94

**4-((6-(Diethylamino)hexyl)oxy)benzaldehyde (2f).** The crude was prepared according to **general procedure B** starting from 4-((6-bromohexyl)oxy)benzaldehyde **1b** (1 equiv., 2.02 mmol, 500 mg), commercially available diethylamine (2 equiv., 4.04 mmol, 0.416 mL) and K_2_CO_3_ (3 Equiv., 6.06 mmol, 837.55 mg) in acetonitrile (20 mL) to afford 237.4 mg of the desired compound (42%) as a transparent oil. The crude was used without further purifications. ^1^H NMR (400 MHz, CDCl_3_) δ 9.87 (s, 1H), 7.85–7.78 (m, 2H), 7.03–6.92 (m, 2H), 4.03 (t, J = 6.5 Hz, 2H), 2.54 (q, J = 7.2 Hz, 5H), 2.45–2.36 (m, 2H), 1.82 (td, J = 13.3, 6.6 Hz, 3H), 1.56–1.43 (m, 4H), 1.39–1.31 (m, 2H), 1.03 (t, J = 7.2 Hz, 7H). ^13^C NMR (101 MHz, CDCl_3_) δ 190.82, 164.24, 132.00, 129.77, 114.76, 68.33, 52.82, 29.06, 27.44, 26.90, 25.98, 11.58

**4-((6-(4-Methylpiperazin-1-yl)hexyl)oxy)benzaldehyde (2g).** The crude was prepared according to **general procedure B** starting from 4-((6-bromohexyl)oxy)benzaldehyde **1b** (1 equiv., 4.03 mmol, 1 g), commercially available N-methylpiperazine (2 equiv., 8.06 mmol, 0.894 mL) and K_2_CO_3_ (3 Equiv., 12.09 mmol, 1.67 g) in acetonitrile (40 mL) to afford 632 mg of the desired compound (49%) as a transparent oil. The crude was used without further purifications. ^1^H NMR (400 MHz, CDCl_3_) δ 9.85 (s, 1H), 7.87–7.68 (m, 2H), 6.99–6.91 (m, 2H), 4.01 (t, *J* = 6.5 Hz, 2H), 2.44 (bs, 8H), 2.36–2.30 (m, 2H), 2.26 (s, 3H), 1.94–1.71 (m, 2H), 1.55–1.41 (m, 4H), 1.36 (td, *J* = 8.6, 1.4 Hz, 2H). ^13^C NMR (101 MHz, CDCl_3_) δ 190.84, 164.21, 132.00, 129.76, 114.7, 68.26, 58.59, 55.02, 53.14, 45.98, 28.99, 27.30, 26.75, 25.92.

**4-((6-(4-Benzylpiperidin-1-yl)hexyl)oxy)benzaldehyde (2h).** The crude was prepared according to **general procedure B.** starting 4-((6-bromohexyl)oxy)benzaldehyde **1b** (1 equiv., 2.84 mmol, 705.47 mg), 4-benzylpiperidine (2 equiv., 5.69 mmol, 1.00 mL) and K_2_CO_3_ (3 Equiv., 8.52 mmol, 1177.55 mg) in acetonitrile (28.2 mL) to afford 752 mg of the desired compound as a transparent oil. The crude was used without further purifications.

##### Synthesis of Compounds 3a-p

**Dimethyl-4-(4-((5-(diethylamino)pentyl)oxy)phenyl)-2,6-dimethyl-1,4-dihydropyridine-3,5-dicarboxylate (3d). 3d** was prepared according to **general procedure C** starting from 4-((5-(diethylamino)pentyl)oxy)benzaldehyde **2b** (1 equiv., 0.81 mmol, 214.00 mg), methyl acetoacetate (3.5 equiv., 2.84 mmol, 306.7 mL) and ammonium carbonate (2.5 equiv., 2.03 mmol, 195.00 mg) at 75 °C over 15h. After being worked up, the crude was finally purified by flash column chromatography using DCM/MeOH 90/10 + 1% Et_3_N to afford 104.00 mg (28%) of **3d** as yellow oil. ^1^H NMR (400 MHz, CDCl_3_) δ 7.19–7.10 (m, 2H), 6.77–6.68 (m, 2H), 5.66 (s, 1H), 4.93 (s, 1H), 3.90 (t, J = 6.4 Hz, 2H), 3.64 (d, J = 2.5 Hz, 6H), 2.59 (q, J = 7.1 Hz, 4H), 2.51–2.40 (m, 2H), 2.33 (s, 6H), 1.82–1.70 (m, 2H), 1.59–1.50 (m, 2H), 1.49–1.40 (m, 2H), 1.06 (t, J = 7.2 Hz, 7H). ^13^C NMR (101 MHz, CDCl_3_) δ 168.25, 157.60, 144.08, 139.90, 128.69, 114.05, 104.23, 67.79, 52.84, 51.09, 46.97, 38.51, 29.82, 29.41, 26.66, 24.32, 19.70, 11.54. HRMS ESI-TOF [M]^+^ m/z calcd. for C_26_H_38_N_2_O_5_: 458.2770, found: 458.2781.

**Diethyl-4-(4-((5-(diethylamino)pentyl)oxy)phenyl)-2,6-dimethyl-1,4-dihydropyridine-3,5-dicarboxylate (3c). 3c** was prepared according to **general procedure C** starting from 4-((5-(diethylamino)pentyl)oxy)benzaldehyde **2b** (1 equiv., 1.52 mmol, 400.00 mg), ethyl acetoacetate (3.5 Equiv., 5.32 mmol, 520.00 mL) and ammonium carbonate (2.5 equiv., 3.80 mmol, 365.14 mg) at 75 °C over 15h. After being worked up, the crude was finally purified by flash column chromatography using DCM/MeOH 90/10 + 1% Et_3_N to afford 54.00 mg (7%) of **3c** as colorless oil. ^1^H NMR (400 MHz, CDCl_3_) δ 7.19–7.14 (m, 2H), 6.76–6.69 (m, 2H), 5.60 (s, 1H), 4.91 (s, 1H), 4.14–4.02 (m, 4H), 3.90 (t, J = 6.4 Hz, 2H), 2.61 (dd, J = 14.2, 7.1 Hz, 4H), 2.55–2.45 (m, 2H), 2.32 (s, 6H), 1.85–1.71 (m, 2H), 1.56 (dt, J = 10.1, 7.4 Hz, 2H), 1.45 (dd, J = 15.1, 8.0 Hz, 2H), 1.22 (t, J = 7.1 Hz, 6H), 1.07 (t, J = 7.1 Hz, 6H). ^13^C NMR (101 MHz, CDCl_3_) δ 167.83, 157.51, 143.64, 140.33, 129.08, 113.88, 104.56, 67.76, 59.81, 52.71, 46.95, 38.86, 29.36, 26.34, 24.28, 19.74, 14.41, 11.28. HRMS ESI-TOF [M]^+^ m/z calcd. for C_28_H_42_N_2_O_5_: 486,3071, found: 486,3094.

**Dimethyl-2,6-dimethyl-4-(4-((5-morpholinopentyl)oxy)phenyl)-1,4-dihydropyridine-3,5-dicarboxylate (3b). 3b** was prepared according to **general procedure C** starting from 4-((5-morpholinopentyl)oxy)benzaldehyde **2a** (1 equiv., 1.49 mmol, 413.00 mg), methyl acetoacetate (3.5 equiv., 5.96 mmol, 643.00 mL) and ammonium carbonate (2.5 equiv., 4.47 mmol, 429.00 mg) at 75 °C over 15h. After being worked up, the crude was finally purified by flash column chromatography using DCM/MeOH 97/3 + 1% Et_3_N to afford 147.00 mg (21%) of **3b** as yellow oil. ^1^H NMR (400 MHz, CDCl_3_) δ 7.19–7.10 (m, 2H), 6.78–6.66 (m, 2H), 5.72 (s, 1H), 4.93 (s, 1H), 3.89 (t, J = 6.4 Hz, 2H), 3.76–3.67 (m, 4H), 3.63 (s, 6H), 2.46 (bs, 4H), 2.40–2.33 (m, 2H), 2.32 (s, 6H), 1.81–1.72 (m, 2H), 1.61–1.51 (m, 2H), 1.50–1.41 (m, 2H). ^13^C NMR (101 MHz, CDCl_3_**)** δ 168.22, 157.32, 143.99, 140.24, 128.83, 114.05, 104.31, 67.08, 63.73, 58.08, 52.04, 51.14, 38.60, 28.72, 23.71, 19.79. Anal. Calcd. for C_26_H_36_N_2_O_6_: C, 66.08; H, 7.68; N, 5.93; found: C, 64.78; H, 7.81; N, 5.98.

**Diethyl-2,6-dimethyl-4-(4-((5-morpholinopentyl)oxy)phenyl)-1,4-dihydropyridine-3,5-dicarboxylate (3a). 3a** was prepared according to **general procedure C** starting from 4-((5-morpholinopentyl)oxy)benzaldehyde **2a** (1 equiv., 0.927 mmol, 257.00 mg), ethyl acetoacetate (3.5 equiv., 3.71 mmol, 473.00 mL) and ammonium carbonate (2.5 equiv., 2.78 mmol, 267.00 mg) at 75 °C over 15h. After being worked up, the crude was finally purified by flash column chromatography using DCM/MeOH 95/5 + 1% Et_3_N to afford 223.00 mg (48%) of **3a** as a yellow oil. ^1^H NMR (400 MHz, CDCl_3_) δ 7.21–7.08 (m, 2H), 6.78–6.65 (m, 2H), 5.57 (s, 1H), 4.92 (s, 1H), 4.21–4.00 (m, 4H), 3.90 (t, J = 6.4 Hz, 2H), 3.77 (bs, 4H), 2.51 (bs, 4H), 2.42 (bs, 2H), 2.32 (s, 6H), 1.88–1.68 (m, 2H), 1.60 (bs, 2H), 1.55–1.40 (m, 2H), 1.22 (t, J = 7.1 Hz, 6H). ^13^C NMR (101 MHz, CDCl_3_) δ 167.83, 157.48, 143.71, 140.34, 129.04, 113.84, 104.46, 67.69, 66.99, 59.78, 59.07, 53.83, 38.84, 29.33, 26.31, 24.13, 19.65, 14.39. Anal. Calcd. for C_28_H_40_N_2_O_6_: C, 67.18; H, 8.05; N, 5.60; found: C, 66.01; H, 8.15; N, 5.79.

**Dimethyl-2,6-dimethyl-4-(4-((5-(4-methylpiperazin-1-yl)pentyl)oxy)phenyl)-1,4-dihydropyridine-3,5-dicarboxylate (3f). 3f** was prepared according to **general procedure C** starting from 4-((5-(4-methylpiperazin-1-yl)pentyl)oxy) benzaldehyde **2c** (1 equiv., 0.837 mmol, 243.00 mg), methyl acetoacetate (3.5 equiv., 3.35 mmol, 362.00 mL) and ammonium carbonate (2.5 equiv., 2.51 mmol, 241.00 mg) at 75 °C over 15h. After being worked up, the crude was finally purified by flash column chromatography using DCM/MeOH 92/8 + 1% Et_3_N to afford 46.05 mg (11%) of **3f** as a yellow oil. ^1^H NMR (400 MHz, CDCl_3_) δ 7.17–7.10 (m, 2H), 6.72 (d, J = 8.6 Hz, 2H), 5.89 (s, 1H), 4.92 (s, 1H), 4.08 (t, J = 6.5 Hz, 2H), 3.89 (t, J = 6.3 Hz, 2H), 3.63 (s, 6H), 3.53–3.47 (m, 4H), 2.38 (t, J = 2.4 Hz, 4H), 2.31 (d, J = 1.9 Hz, 9H), 1.81–1.72 (m, 2H), 1.72–1.63 (m, 2H), 1.56–1.46 (m, 2H). ^13^C NMR (101 MHz, CDCl_3_) δ 168.26, 157.52, 155.58, 144.17, 140.00, 128.68, 114.02, 104.12, 67.57, 65.52, 54.74, 51.08, 46.13, 43.55, 38.49, 29.09, 28.85, 22.75, 19.63. HRMS ESI-TOF [M]^+^ m/z calcd. for C_27_H_39_N_3_O_5_: 485,2869, found: 485,2890.

**Diethyl-2,6-dimethyl-4-(4-((5-(4-methylpiperazin-1-yl)pentyl)oxy)phenyl)-1,4-dihydropyridine-3,5-dicarboxylate (3e). 3e** was prepared according to **general procedure C** starting from 4-((5-(4-methylpiperazin-1-yl)pentyl)oxy) benzaldehyde **2c** (1 equiv., 0.689 mmol, 200.00 mg), ethyl acetoacetate (3.5 equiv., 2.40 mmol, 308.12 mL) and ammonium carbonate (2.5 equiv., 1.72 mmol, 165.00 mg) at 75 °C over 15h. After being worked up, the crude was finally purified by flash column chromatography using DCM/MeOH 98/2 + 1% Et_3_N to afford 114.7 mg (32%) of **3e** as a light brown solid. ^1^H NMR (400 MHz, CDCl_3_) δ 7.22–7.09 (m, 2H), 6.79–6.62 (m, 2H), 5.54 (s, 1H), 4.92 (s, 1H), 4.18–4.00 (m, 4H), 3.89 (t, J = 6.4 Hz, 2H), 2.59 (bs, 8H), 2.45–2.39 (m, 2H), 2.36 (s, 3H), 2.32 (s, 6H), 1.81–1.71 (m, 2H), 1.63–1.53 (m, 2H), 1.50–1.40 (m, 2H), 1.22 (t, J = 7.1 Hz, 6H). ^13^C NMR (101 MHz, CDCl_3_) δ 167.82, 157.51, 143.58, 140.33, 129.09, 113.88, 104.61, 77.48, 77.16, 76.84, 67.68, 59.83, 58.43, 52.73, 45.78, 38.88, 29.32, 24.18, 19.78, 14.43. HRMS ESI-TOF [M]^+^ m/z calcd. for C_29_H_43_N_3_O_5_: 513,3197, found: 513,3203.

**Dimethyl-4-(4-((5-(4-benzylpiperidin-1-yl)pentyl)oxy)phenyl)-2,6-dimethyl-1,4-dihydropyridine-3,5-dicarboxylate (3h). 3h** was prepared according to **general procedure C** starting from 4-((5-(4-benzylpiperidin-1-yl)pentyl)oxy)benzaldehyde **2d** (1 equiv., 0.783 mmol, 286.00 mg), methyl acetoacetate (3.5 equiv., 3.13 mmol, 338.10 mL) and ammonium carbonate (2.5 equiv., 2.35 mmol, 226.00 mg) at 75 °C over 15h. After being worked up, the crude was finally purified by flash column chromatography using DCM/MeOH 98/2 + 1% Et_3_N to afford 64.32 mg (15%) of **3h** as ayellow oil. ^1^H NMR (400 MHz, CDCl_3_) δ 7.30–7.23 (m, 4H), 7.20–7.06 (m, 5H), 6.75–6.65 (m, 2H), 5.76–5.67 (m, 1H), 4.93 (s, 1H), 3.88 (t, J = 6.5 Hz, 2H), 3.63 (d, J = 2.5 Hz, 6H), 2.92 (d, J = 11.4 Hz, 2H), 2.53 (d, J = 7.0 Hz, 2H), 2.36–2.27 (m, 6H), 1.87 (t, J = 11.2 Hz, 2H), 1.79–1.70 (m, 2H), 1.63 (d, J = 12.1 Hz, 2H), 1.58–1.48 (m, 4H), 1.46–1.39 (m, 3H), 1.37–1.30 (m, 2H). ^13^C NMR (101 MHz, CDCl_3_) δ 168.23, 157.59, 144.06, 140.80, 139.88, 129.23, 128.68, 128.27, 125.89, 114.03, 104.21, 67.73, 59.06, 54.07, 51.08, 43.29, 38.50, 38.03, 32.14, 29.36, 26.81, 24.34, 19.68. HRMS ESI-TOF [M]^+^ m/z calcd. for C_34_H_44_N_2_O_5_: 560,3238, found: 560,3250.

**Diethyl-4-(4-((5-(4-benzylpiperidin-1-yl)pentyl)oxy)phenyl)-2,6-dimethyl-1,4-dihydropyridine-3,5-dicarboxylate (3g). 3g** was prepared according to **general procedure C** starting from 4-((5-(4-benzylpiperidin-1-yl)pentyl)oxy)-benzaldehyde **2d** (1 equiv., 1.88 mmol, 687.00 mg), ethyl acetoacetate (3.5 equiv., 7.52 mmol, 1.03 mL) and ammonium carbonate (2.5 Equiv., 5.64 mmol, 542.14 mg) at 75 °C over 15h. After being worked up, the crude was finally purified by flash column chromatography using DCM/MeOH 97/3 + 1% Et_3_N to afford 227.00 mg (21%) of **3g** as yellow oil. ^1^H NMR (400 MHz, CDCl_3_) δ 7.27 (d, J = 7.8 Hz, 1H), 7.24 (s, 1H), 7.15 (dt, J = 8.1, 6.6 Hz, 5H), 6.74–6.67 (m, 2H), 5.88 (bs, 1H), 4.91 (s, 1H), 4.14–4.00 (m, 4H), 3.88 (t, J = 6.4 Hz, 2H), 2.95 (d, J = 11.3 Hz, 2H), 2.53 (d, J = 7.0 Hz, 2H), 2.38–2.32 (m, 2H), 2.31 (s, 6H), 1.91 (t, J = 11.2 Hz, 2H), 1.79–1.70 (m, 2H), 1.64 (d, J = 13.0 Hz, 2H), 1.60–1.48 (m, 3H), 1.47–1.34 (m, 4H), 1.21 (t, J = 7.1 Hz, 6H). ^13^C NMR (101 MHz, CDCl_3_) δ 167.83, 157.42, 143.82, 140.63, 140.32, 129.17, 128.98, 128.23, 125.87, 113.79, 104.30, 77.48, 77.16, 76.84, 67.66, 59.72, 58.90, 53.94, 43.16, 38.78, 37.88, 31.88, 29.27, 26.56, 24.25, 19.54, 14.35. HRMS ESI-TOF [M]^+^ m/z calcd. for C_36_H_48_N_2_O_5_: 588,3535, found: 588,3563.

**3,5-Dimethyl-4-(4-{[6-(diethylamino)hexyl]oxy}phenyl)-2,6–dimethyl-1,4–dihydropyridine-3,5- dicarboxylate (3l). 3l** was prepared according to **general procedure C** starting from 4-((6-(diethylamino)hexyl)oxy)benzaldehyde **2f** (1 equiv., 0.55 mmol, 160.7 mg), methyl acetoacetate (3.5 equiv., 1.93 mmol, 0.279 mL) and ammonium carbonate (2.5 equiv., 1.38 mmol, 132.60 mg) at 75 °C over 15h. After being worked up, the crude was finally purified by flash column chromatography using AE/MeOH 96/4 + 1% Et_3_N to afford 25.10 mg (3%) of **3l** as yellow oil. ^1^H NMR (400 MHz, CDCl_3_) δ 7.19–7.11 (m, 2H), 6.72 (d, J = 8.6 Hz, 2H), 5.79 (s, 1H), 4.92 (s, 1H), 3.88 (t, J = 6.4 Hz, 2H), 2.69 (dd, J = 14.0, 6.9 Hz, 4H), 2.63–2.50 (m, 2H), 2.33 (s, 6H), 1.75 (dd, J = 14.0, 7.2 Hz, 2H), 1.62–1.53 (m, 2H), 1.50–1.41 (m, 2H), 1.36 (dd, J = 14.9, 7.8 Hz, 2H), 1.12 (t, J = 7.2 Hz, 6H). ^13^C NMR (101 MHz, CDCl_3_) δ 168.24, 157.59, 144.04, 139.94, 128.73, 114.05, 104.28, 67.67, 51.11, 46.84, 38.54, 29.35, 27.28, 26.06, 19.76. HRMS ESI-TOF [M]^+^ m/z calcd. for C_27_H_40_N_2_O_5_: 472,2927, found: 472,2937.

**3,5-Diethyl-4-(4-{[6-(diethylamino)hexyl]oxy}phenyl)–2,6–dimethyl-1,4–dihydropyridine-3,5-dicarboxylate (3k). 3k** was prepared according to **general procedure C** starting from 4-((6-(diethylamino)hexyl)oxy)benzaldehyde **2f** (1 equiv., 0.90 mmol, 249 mg), ethyl acetoacetate (3 equiv., 2.69 mmol, 0.263 mL) and ammonium carbonate (2 equiv., 1.80 mmol, 172.58 mg) at 70 °C over 15h. After being worked up, the crude was finally purified by flash column chromatography using AE/MeOH 98/2 + 1% Et_3_N to afford 129.2 mg (25%) of **3k** as yellow oil. ^1^H NMR (400 MHz, CDCl_3_) δ 7.20–7.10 (m, 2H), 6.77–6.67 (m, 2H), 5.87 (s, 1H), 4.91 (s, 1H), 4.13–3.98 (m, 4H), 3.88 (t, J = 6.4 Hz, 2H), 2.67 (q, J = 7.2 Hz, 4H), 2.55 (dd, J = 9.3, 6.7 Hz, 2H), 2.31 (s, 6H), 1.78–1.68 (m, 2H), 1.50 (dddd, J = 21.2, 15.5, 10.0, 7.3 Hz, 5H), 1.38–1.29 (m, 2H), 1.21 (t, J = 7.1 Hz, 6H), 1.08 (t, J = 7.2 Hz, 6H). ^13^C NMR (101 MHz, CDCl_3_) δ 167.81, 157.55, 143.58, 140.28, 129.08, 113.89, 104.61, 67.78, 59.83, 46.93, 38.88, 29.43, 27.46, 26.15, 19.79, 14.43. HRMS ESI-TOF [M]^+^ m/z calcd. for C_29_H_44_N_2_O_5_: 500,3236, found: 500,3250.

**3,5-Dimethyl-2,6-dimethyl-4-(4-{[6-(morpholin-4-yl)hexyl]oxy}phenyl)-1,4-dihydropyridine-3,5-dicarboxylate (3j). 3j** was prepared according to **general procedure C** starting from 4-((6-morpholinohexyl)oxy)benzaldehyde **2e** (1 equiv., 0.69 mmol, 200 mg), methyl acetoacetate (3.5 equiv., 2.40 mmol, 0.259 mL) and ammonium carbonate (2.5 equiv., 1.72 mmol, 165.03 mg) at 60 °C over 15h. After being worked up, the crude was finally purified by flash column chromatography using DCM/MeOH 95/5 + 1% NH_3_ to afford 53 mg (7%) of **3j** as birght orange oil. ^1^H NMR (400 MHz, CDCl_3_) δ 7.15 (d, J = 8.7 Hz, 2H), 6.73 (d, J = 8.7 Hz, 2H), 5.63 (s, J = 14.2 Hz, 1H), 4.93 (s, J = 4.1 Hz, 1H), 3.89 (t, J = 6.4 Hz, 2H), 3.80 (s, 4H), 3.64 (d, J = 2.4 Hz, 6H), 2.66–2.51 (m, J = 9.6, 7.8 Hz, 4H), 2.47 (bs, J = 15.5 Hz, 2H), 2.33 (s, 6H), 1.80–1.68 (m, 2H), 1.60 (bs, 2H), 1.52–1.42 (m, 2H), 1.41–1.31 (m, 2H). ^13^C NMR (101 MHz, CDCl_3_) δ 168.23, 157.61, 143.99, 139.92, 128.91, 128.73, 114.06, 113.97, 104.31, 67.70, 58.99, 53.52, 51.12, 38.55, 29.35, 27.23, 26.09, 19.77. HRMS ESI-TOF [M]^+^ m/z calcd. for C_27_H_38_N_2_O_6_: 486,2711, found: 486,2730.

**3,5-Ethyl-2,6-dimethyl-4-(4-{[6-(morpholin-4-yl)hexyl]oxy}phenyl)-1,4-dihydropyridine-3,5-dicarboxylate (3i). 3i** was prepared according to **general procedure C** starting from 4-((6-morpholinohexyl)oxy)benzaldehyde **2e** (1 equiv., 0.412 mmol, 120 mg), ethyl acetoacetate (2.5 equiv., 1.03 mmol, 0.089 mL) and ammonium carbonate (1.5 equiv., 0.62 mmol, 59.38 mg) at 70 °C over 15h. After being worked up, the crude was finally purified by flash column chromatography using DCM/MeOH 96/4 + 1% NH_3_ to afford 37.00 mg (19%) of **3i** as a colorless oil. ^1^H NMR (400 MHz, CDCl_3_) δ 7.21–7.12 (m, 2H), 6.77–6.69 (m, 2H), 5.54 (s, 1H), 4.92 (s, 1H), 4.16–4.02 (m, 4H), 3.89 (t, J = 6.4 Hz, 2H), 3.76 (bs, 4H), 2.49 (bs, 4H), 2.38 (bs, 2H), 2.32 (s, 6H), 1.80–1.68 (m, 2H), 1.50–1.41 (m, 3H), 1.40–1.31 (m, 3H), 1.22 (t, J = 7.1 Hz, 6H). ^13^C NMR (101 MHz, CDCl_3_) δ 167.82, 157.55, 143.57, 140.31, 129.09, 113.89, 104.61, 67.77, 59.83, 59.11, 53.69, 38.88, 29.40, 27.32, 26.14, 19.78, 14.42. HRMS ESI-TOF [M]^+^ m/z calcd. for C_29_H_42_N_2_O_6_: 514,3028, found: 514,3043.

**3,5-Dimethyl-2,6-dimethyl-4-(4-{[6-(4-methylpiperazin-1-yl)hexyl]oxy}phenyl)-1,4-dihydro-pyridine-3,5-dicarboxylate (3n). 3n** was prepared according to **general procedure C** starting from 4-((6-(4-methylpiperazin-1-yl)hexyl)oxy)benzaldehyde **2g** (1 equiv., 0.82 mmol, 250 mg), methyl acetoacetate (4 equiv., 3.29 mmol, 0.355 mL) and ammonium carbonate (3 equiv., 2.46 mmol, 236.38 mg) at 70 °C over 15h. After beingworked up, the crude was finally purified by flash column chromatography using EA/MeOH 95/5 + 1% Et_3_N to afford 61.6 mg of **3n** as white powder (15%). ^1^H NMR (400 MHz, CDCl_3_) δ 7.15 (d, J = 8.6 Hz, 2H), 6.72 (d, J = 8.6 Hz, 2H), 5.66 (s, 1H), 4.93 (s, 1H), 3.88 (t, J = 6.4 Hz, 2H), 3.64 (sJ, 6H), 2.54 (bs, 8H), 2.43–2.36 (m, 2H), 2.33 (s, 9H), 1.80–1.66 (m, 2H), 1.59–1.50 (m, 2H), 1.48–1.39 (m, 2H), 1.35 (dd, J = 14.0, 7.0 Hz, 2H). ^13^C NMR (101 MHz, CDCl_3_) δ 168.23, 157.63, 144.01, 139.88, 128.71, 114.06, 104.29, 67.76, 58.60, 54.86, 52.95, 51.11, 45.92, 38.52, 29.83, 29.39, 27.40, 26.70, 26.14, 19.76. Anal. Calcd. for C_28_H_41_N_3_O_5_: C, 64.48; H, 8.27; N, 8.41; found: C, 65.48; H, 8.31; N, 8.53. HRMS ESI-TOF [M]^+^ m/z calcd. for C_28_H_41_N_3_O_5_: 499,3039, found: 499,3046.

**3,5-Diethyl-2,6-dimethyl-4-(4-{[6-(4-methylpiperazin-1-yl)hexyl]oxy}phenyl)-1,4-dihydro-pyridine-3,5-dicarboxylate (3m). 3m** was prepared according to **general procedure C** starting from 4-((6-(4-methylpiperazin-1-yl)hexyl) oxy)benzaldehyde **2g** (1 equiv., 0.70 mmol, 212 mg), ethyl acetoacetate (3 equiv., 2.09 mmol, 0.204 mL) and ammonium carbonate (2 equiv., 1.39 mmol, 133.95 mg) at 70 °C over 15h. After was worked up, the crude was finally purified by flash column chromatography using EA/MeOH 97/3 + 1% Et_3_N to afford 144.4 mg (20%) of **3m** as a yellow oil. ^1^H NMR (400 MHz, CDCl_3_) δ 7.16 (d, J = 8.7 Hz, 2H), 6.71 (d, J = 8.7 Hz, 2H), 5.62 (s, 1H), 4.91 (s, 1H), 4.14–3.99 (m, 4H), 3.88 (t, J = 6.4 Hz, 2H), 2.59 (bs, 8H), 2.49–2.39 (m, 2H), 2.36 (s, 3H), 2.32 (s, 6H), 1.78–1.68 (m, 2H), 1.60–1.50 (m, 2H), 1.50–1.41 (m, 2H), 1.40–1.31 (m, 2H), 1.22 (t, J = 7.1 Hz, 6H). ^13^C NMR (101 MHz, CDCl_3_) δ 167.82, 157.53, 143.63, 140.31, 129.07, 113.88, 104.56, 67.74, 59.81, 58.41, 54.51, 52.60, 45.69, 38.86, 29.37, 27.31, 26.09, 19.76, 14.42. HRMS ESI-TOF [M]^+^ m/z calcd. for C_30_H_45_N_3_O_5_: 527,3341, found: 527,3359.

**3,5-Dimethyl-4-(4-{[6-(4-benzylpiperidin-1-yl)hexyl]oxy}phenyl)-2,6-dimethyl-1,4-dihydro-pyridine-3,5-dicarboxylate (3p). 3p** was prepared according to **general procedure C** starting from 4-((6-(4-benzylpiperidin-1-yl)hexyl)oxy)benzaldehyde **2h** (1 equiv., 0.79 mmol, 300 mg), methyl acetoacetate (3 equiv., 2.37 mmol, 0.256 mL) and ammonium carbonate (2 equiv., 1.58 mmol, 152.02 mg) at 70 °C over 24h. After being worked up, the crude was finally purified by flash column chromatography using DCM/MeOH 92/8 + 1% NH_3_ to afford 29.91 mg (7%) of **3p** as light yellow oil. ^1^H NMR (400 MHz, CDCl_3_) δ 7.30–7.26 (m, 1H), 7.25 (s, 1H), 7.22–7.11 (m, 5H), 6.79–6.66 (m, 2H), 5.72 (d, J = 13.5 Hz, 1H), 4.93 (s, 1H), 3.87 (t, J = 6.4 Hz, 2H), 3.64 (s, 6H), 3.03 (bs, 2H), 2.55 (d, J = 6.3 Hz, 2H), 2.42 (bs, 2H), 2.33 (s, 6H), 2.02 (bs, 2H), 1.77–1.63 (m, 5H), 1.63–1.52 (m, 4H), 1.49–1.40 (m, 3H), 1.34 (dd, J = 13.7, 6.2 Hz, 2H). ^13^C NMR (101 MHz, CDCl_3_) δ 168.25, 157.47, 144.12, 140.06, 129.14, 128.93, 128.75, 128.66, 126.53, 114.03, 113.94, 104.21, 67.39, 59.84, 57.72, 53.12, 51.10, 42.12, 38.54, 36.84, 29.12, 29.03, 26.72, 25.71, 23.63, 19.73. HRMS ESI-TOF [M]^+^ m/z calcd. for C_35_H_46_N_2_O_5_: 574,3391, found: 574,3407.

**3,5-Dimethyl-4-(4-{[6-(4-benzylpiperidin-1-yl)hexyl]oxy}phenyl)-2,6-dimethyl-1,4-dihydro-pyridine-3,5-dicarboxylate (3o). 3o** was prepared according to **general procedure C** starting from 4-((6-(4-benzylpiperidin-1-yl)hexyl)oxy)benzaldehyde **2h** (1 equiv., 0.79 mmol, 300 mg), ethyl acetoacetate (2.5 equiv., 1.97 mmol, 0.251 mL) and ammonium carbonate (1.5 equiv., 1.19 mmol, 114.01 mg) at 75 °C over 20h. The crudewas dissolved in MeOH and solvent was evaporated in vacuo. The residue was dissolved in DCM and washed with water and brine. The organic layer was then dried over Na_2_SO_4_ and finally purified by flash column chromatography using DCM/MeOH 96/4 + 1% NH_3_ to afford 144.4 mg (31%) of **3o** as dark yellow oil. ^1^H NMR (400 MHz, CDCl_3_) δ 7.30–7.26 (m, 1H), 7.25 (s, 1H), 7.21–7.10 (m, 5H), 6.76–6.64 (m, 2H), 5.68 (s, 1H), 4.91 (s, 1H), 4.14–4.03 (m, 4H), 3.88 (t, J = 6.4 Hz, 2H), 2.95 (d, J = 11.2 Hz, 2H), 2.53 (d, J = 7.0 Hz, 2H), 2.32 (s, 7H), 1.90 (t, J = 11.2 Hz, 2H), 1.78–1.69 (m, 2H), 1.64 (d, J = 12.8 Hz, 2H), 1.58–1.49 (m, 4H), 1.47–1.29 (m, 7H), 1.22 (t, J = 7.1 Hz, 6H). ^13^C NMR (101 MHz, CDCl_3_) δ 167.82, 157.55, 143.66, 140.77, 140.27, 129.24, 129.05, 128.28, 125.91, 113.87, 104.52, 67.81, 59.79, 59.11, 43.27, 38.84, 38.01, 32.05, 29.41, 27.57, 26.89, 26.15, 19.70, 14.40. Anal. Calcd. for C_37_H_50_N_2_O_5_: C, 73.72; H, 8.36; N, 4.65; found: C, 71.09; H, 8.47; N, 4.58. HRMS ESI-TOF [M]^+^ m/z calcd. for C_37_H_50_N_2_O_5_: 602,3705, found: 602,3720.

### 3.2. Biological Evaluation

#### 3.2.1. Inhibition of EeAChE and eqBuChE

The evaluation of the activity of compounds against both EeAChE and eqBuChE was evaluated following the Ellman [22] spectrophotometric method. Briefly, the reaction happens in a final volume of 3 mL of a 0.1 M phosphate-buffered solution at pH = 8.0, containing 5,5′-dithiobis-2-nitrobenzoic acid, EeAChE or eqBuChE, tested compound and bovine albumin serum phosphate-buffered (pH = 8) solution After this pre-incubation period, acetylthiocholine iodide or butyrylthiocholine iodide was added, allowing 15 min of additional incubation time. For IC_50_, inhibition curves were built by pre-incubating this blend at room temperature with nine concentrations of each compound for 10 min [28].

#### 3.2.2. Calcium Channel Blockade

The calcium channel blockade assay was performed as described earlier [29]. Briefly, the SH-SY5Y cells were seeded out in 96-well dark-walled plates at a density of 1 × 10^5^ cells per well. After 24 h, the cells were loaded with FLIPR Calcium 6 indicator (Molecular Devices) for 2 h at 37 °C, according to the manufacturer’s protocol. Compounds were dissolved in an appropriate amount of DMSO at 10 mM concentration and diluted to a final concentration of 10 μM in Hanks′ Balanced Salt Solution (HBSS, Thermo Fisher Scientific) buffered with HEPES (Sigma-Aldrich) at pH = 7.4, and as such used were for treatment of indicator loaded cells (10 min at 37 °C). The fluorescence of indicator-loaded cells was measured with Synergy H1 (Biotek Instruments, USA) multilabel plate reader at excitation at 485 nm and emission at 525 nm wavelengths. The baseline fluorescence was recorded for 5 s. Then, the cells were stimulated with KCl/CaCl_2_ solution (in HBSS, final concentration of KCl and CaCl_2_ was 90 and 5 mM, respectively) and the fluorescence was recorded for a further 30 s. Dimethyl sulfoxide 1% solution in HBSS was used as a vehicle control. Nimodipine (Cayman Chemical Company) was used as a reference inhibitor at 10 μM and compounds were assessed at the same final concentration in triplicate in three independent experiments. Fluorescent intensity values were normalized to the baseline.

#### 3.2.3. Oxygen Radical Absorbance Capacity Assay

The antioxidant activity of compounds **3a**-**p** was performed by the ORAC-FL using fluorescein as a fluorescent probe. Briefly, fluorescein and antioxidant were incubated in a black 96-well microplate (Nunc) for 15 min at 37 °C. 2,2′-Azobis(amidinopropane) dihydrochloride was then added quickly using the built-in injector of Varioskan Flash plate reader (Thermo Scientific). The fluorescence was measured at 485 nm (excitation wavelength) and 535 nm (emission wavelength) each min for 1 h. All the reactions were made in triplicate and at least three different assays were performed for each sample [9,30].

#### 3.2.4. Effect of Compounds 3c, 3h and 3m on H_2_O_2_ on a mixture of Oligomycin A/Rotenone-induced cell death in SH-SY5Y cells

SH-SY5Y cells were seeded in 96-well culture plates at a density of 12 × 10^4^ cells per well in DMEM/F12 (1:1) medium supplemented with 10% fetal bovine serum, 1X non-essential amino acids, 100 units/mL penicillin and 10 mg/mL streptomycin (Dutscher, France). After 48 h of incubation, the cultures were treated with 100 µl of the test compounds or DMSO (0.1%) in the same medium. After 24 h, the cells were co-incubated with H_2_O_2_ (200 µM) or O/R (O at 10 µM/R at 30 µM) with or without the tested compounds at noncytotoxic concentrations for an additional period of 24 h. The percentage of cell viability was measured using CellTiter 96 AQueous Non-Radioactive Cell Proliferation (MTS) Assay (Promega, France).

### 3.3. Molecular Modelling

Compounds **3c**, **3d**, **3h** and **3k-3m** were built using Discovery Studio 2.1 (DS 2.1) and protonated on a piperidine ring following the energy-minimization using the adopted-based Newton–Raphson algorithm. The protein crystal structure of hAChE in complex with fasciculine (PDB ID: 1B41) was prepared prior to docking, using the protein model tool in Discovery Studio. Cocrystal ligands and water molecules were removed and AutoDockTools (ADT; version 1.5.6) was used to add hydrogens and partial charges for proteins and ligands using Gasteiger charges.

To give flexibility to the binding site, residues lining the AChE (Trp286, Tyr124, Tyr337, Tyr341, Tyr72, Asp74, Thr75, Trp86) were allowed to move during the docking search as performed by software Autodock Vina [27]. The grid box was built with a resolution of 1 Å and 60 × 60 × 72 points and it was positioned at the middle of the protein (x = 116.546; y = 110.33; z = −134.181).

For hBuChE, the three-dimensional structure in complex with tacrine has been used (PDB ID: 4BDS). The preparation of this protein was done following the same protocol described before for hAChE. All dockings were performed where a box of 66 × 66 × 70 Ǻ with grid points separated 1 Ǻ, was positioned at the middle of the protein (x = 136.0; y = 123.59; z = 38.56).

The ADT program was used to generate the docking input files. Docking calculations were performed as blind dockings with the program Autodock Vina [27] and default parameters were used, except num_modes, which was set to 40.

The best Vina scored poses were considered and the docked ligand output files were viewed and analyzed using DS.

## 4. Conclusions

AD is a neurodegenerative disorder and is the most common form of senile dementia. The four current AD pharmacotherapies focus on the impairment of cholinergic and glutamatergic systems, but with limited therapeutic success. So, it is urgent to develop new hits to combat AD. For this purpose, compounds **3a**-**g** have been successfully synthesized by Hantzsch multicomponent reaction, and their biological evaluation, as potential cholinesterase inhibitors, Ca^+2^ channel blockers, antioxidant and neuroprotective agents has been assessed. The inhibition of cholinesterase allowed us to identify five compounds, **3c**, **3h** and **3k-l**, as micromolar inhibitors against BuChE and only **3h** as a micromolar inhibitor against AChEI. Concerning Ca^+2^ channel blockade results, it is worth mentioning that compounds **3c**, **3g-j, 3m** and **3o-p** showed inhibitions higher than or comparable with nimodipine. Compounds **3b**-**c** and **3h** showed antioxidant activities higher than melatonin used as standard. To sum up, these biological analyses have prompted us to identify compound **3h** (dimethyl-4-(4-((5-(4-benzylpiperidin-1-yl)pentyl)oxy)phenyl)-2,6-dimethyl-1,4-dihydropyridine-3,5-dicarboxylate) as the most balanced MTDL, showing IC_50_ equal to 1.8 and 2 μM against AChE and BuChE, respectively, and a percentage of Ca^+2^ channel blockade equal to 47.72% at 10 μM with additional interesting antioxidant effect (2.54 TE). The neuroprotective activity results support the interest in compound **3h**, particularly for its neuroprotective activity against H_2_O_2_ (28.68%) and O/R (38.29%) in SHSY5Y cells at 0.3 μM, thus being considered as a new hit-compound for further investigation in our search for anti-AD agents. This work is now in progress in our laboratories, to develop analogues with best pharmacological profile, and the results will be presented elsewhere in due course.

## Figures and Tables

**Table 1:** ChE inhibition and calcium blockade percentages for compounds **2a-p**, and their ORAC (TE) values.

Cpd	Amine	*n*	R	AChE ^a^ IC_50_ (μM)	BuChE ^a^ IC_50_ (μM)	(%) Ca Flux Inhibition ^b^	ORAC ^c^
3a	Morpholine	5	OEt	- ^d^	- ^d^	18.58	1.31 ± 0.01
3b		5	OMe	- ^d^	- ^d^	31.89	2.59 ± 0.04
**3c**	Diethylamine	5	OEt	- ^d^	**4.6 ± 0.1**	**58.21**	**2.64 ± 0.04**
3d		5	OMe	- ^d^	- ^d^	39.55	1.93 ± 0.68
3e	*N*-Me Piperazine	5	OEt	- ^d^	- ^d^	40.51	1.78 ± 0.00
3f		5	OMe	- ^d^	- ^d^	38.4	1.38 ± 0.02
3g	4-Bn Piperidine	5	OEt	- ^d^	- ^d^	53.6	2.24 ± 0.04
**3h**		5	OMe	**1.8 ± 0.1**	**2.0 ± 0.1**	**47.72**	**2.54 ± 0.04**
3i	Morpholine	6	OEt	- ^d^	- ^d^	47.55	1.22 ± 0.02
3j		6	OMe	- ^d^	- ^d^	52.57	0.99 ± 0.09
3k	Diethylamine	6	OEt	- ^d^	9.6 ± 0.3	31.66	1.21 ± 0.12
3l		6	OMe	- ^d^	8.7 ± 0.1	39.74	0.91 ± 0.01
**3m**	*N*-Me Piperazine	6	OEt	- ^d^	**5.5 ± 0.1**	**50.6**	1.10 ± 0.08
3n		6	OMe	- ^d^	- ^d^	28.9	1.39 ± 0.12
**3o**	4-*N*-Bn Piperidine	6	OEt	- ^d^	- ^d^	**80.19**	1.36 ± 0.01
**3p**		6	OMe	- ^d^	- ^d^	**78.13**	2.28 ± 0.01
TA	---			0.03 ± 0.01	5.1 ± 0.1 nM	nd	nd
NIM	---			nd	nd	47.91	nd
MEL	---			nd	nd	nd	2.45 ± 0.09

^a^ Each IC_50_ value is the mean ± SEM of quadruplicate of at least three different experiments^; b^ Every percentage value is the mean of a triple of at least two different experiments. ^c^ Data are expressed as Trolox equivalents and are the mean (*n* = 3) ± SEM. ^d^ % inhibition under 50% at 10 μM. nd: not determined. NIM = nimodipine, MEL = melatonin, TA = tacrine.

**Table 2:** Neuroprotective activity of compounds **3c**, **3h** and **3m** on H_2_O_2_ (200 µM) or oligomycin O at 10 µM)/rotenone (R at 30 µM)-induced cell death in SH-SY5Y cells ^a^.

Compounds	Concentration	H_2_O_2_ (%)	O/R (%)
**3c**	0.3 µM	12.37 ± 0.08	22.27 ± 0.09
	1 µM	24.17 ± 0.12	25.87 ± 0.04
**3h**	0.3 µM	28.68 ± 0.02 *	38.29 ± 0.04 *
	1 µM	23.32 ± 0.02 *	33.90 ± 0.05 *
**3m**	0.3 µM	9.55 ± 0.00	8.95 ± 0.00
	1 µM	4.63 ± 0.02	16.65 ± 0.00
**Melatonin**	0.1 µM	55.4 ± 7.73 *[10]	66.6 ± 3.01 ***[10]

^a^ Data are expressed as % neuroprotection ± SEM of quadruplicates from at least three different cultures; * *p* <  0.0, *** *p*< 0.001 as compared to the control cultures (one-way ANOVA).
